# Priorities for intervention to prevent diarrhea among children aged 0–23 months in northeastern Ethiopia: a matched case-control study

**DOI:** 10.1186/s12887-021-02592-5

**Published:** 2021-03-31

**Authors:** Alemwork Baye, Metadel Adane, Tadesse Sisay, Habtamu Shimels Hailemeskel

**Affiliations:** 1 Neonatal Intensive Care Unit, Dessie Comprehensive Specialized Hospital, Dessie, Ethiopia; 2 Department of Environmental Health, College of Medicine and Health Sciences, Wollo University, Dessie, Ethiopia; 3 Department of Pediatrics and Neonatal Nursing, Health Science College, Debre Tabor University, Debre Tabor, Ethiopia

**Keywords:** Diarrhea, Children aged 0–23 months, Matched case-control study, Northeastern Ethiopia

## Abstract

**Background:**

The global public health problem of diarrhea is most prevalent in developing countries including Ethiopia, especially among children under two years of age. Limited information on the determinants of diarrhea among children aged 0–23 months hinders the design and prioritization of intervention strategies to address childhood diarrhea in Dessie City, northeastern Ethiopia. Therefore, this study was designed to assess the determinants of diarrhea in order to identify priority interventions for its control.

**Methods:**

A community-based matched case-control study was conducted among children aged 0–23 months during January–February 2018. Cases defined as children with acute diarrhea, and controls defined as children without acute diarrhea, were matched by child’s age (months) and place of residence (residing in the same *kebele,* the lowest local administrative unit, each of which has a population of approximately 5000) during the two weeks prior to data collection. Data were collected from mothers/caregivers of the 119 cases and 238 matched controls using a pre-tested structured questionnaire and an observational checklist. Data were analyzed using conditional logistic regression model with 95% confidence interval (CI); variables with *p <* 0.05 from multivariable analysis were considered as significantly associated with acute diarrhea among children aged 0–23 months.

**Results:**

Age of mothers/caregivers (> 35 years of age) (adjusted matched odds ratio [adjusted mOR] = 2.00; 95% CI: 1.37–5.8); divorced/widowed marital status (adjusted mOR = 1.40; 95% CI: 1.26–3.3); lack of exclusive breastfeeding (adjusted mOR = 2.12; 95% CI: 1.15–3.70); presence of feces within/around latrines (adjusted mOR = 1.37; 95% CI: 1.21–3.50); lack of handwashing facility near latrine (adjusted mOR = 1.50; 95% CI: 1.30–5.30); presence of domestic sewage discharge within and/or outside the compound (adjusted mOR = 3.29; 95% CI: 1.85–7.50) and practice of handwashing at fewer than three of the five critical daily times (adjusted mOR = 4.50; 95% CI: 2.54–9.50) were significantly associated with acute diarrhea among children aged under two years.

**Conclusion:**

To reduce acute diarrheal disease among children under two, priority should be given to interventions that focus on improving exclusive breastfeeding practices, regular cleaning of latrines, advocating for availability of handwashing facility within/around latrines, use of proper domestic sewage discharge methods and improving handwashing practice at the five critical times each day. Strengthening communication that promotes hygiene and behavioural change may also raise awareness among mothers/caregivers and empower them to enhance handwashing practices at critical times.

## Introduction

Children under five in urban areas are highly vulnerable to communicable diseases [1]; and for those in low- and middle-income countries, one of the leading public health problems is diarrhea [2], defined as the presence of an abrupt onset of three or more loose or liquid stools within a 24-h period with abdominal symptoms such as cramping, bloating, gas and dehydration [3–5].

Globally, diarrhea is the second leading killer of children under five after pneumonia [6], although its occurrence was reduced from 1.2 million cases in 2000 to 526,000 in 2015 [7]. In developing countries, children under three years of age experience on average three episodes of diarrhea every year; moreover, sub-Saharan Africa continues to have the highest rates of child mortality. Africa and South Asia are where more than 80% of child deaths due to diarrhea occur [7]. Various studies in developing countries have found several factors significantly associated with diarrhea among under-five children, such as unsafe water storage practices [8], lack of household-level water treatment [9], improper disposal of child feces [10], poor nutritional status [11], type of toilet, and place of residence [12].

Furthermore, in Ethiopia, diarrhea is one of the leading causes of mortality and morbidity among children under five. According to the 2016 Ethiopia Demographic and Health Survey (EDHS), the prevalence of diarrhea among children under five was estimated to be 12.0% [13]. A systematic review and meta-analysis study in 2018 revealed that a pooled prevalence of diarrhea among Ethiopian children under five was 22.0% [14]. A study in urban areas of Ethiopia revealed that inadequate sanitation facilities and poor hygiene practices [15], interrupted water supply [16] and poor hand washing practice [17] were determinants of acute diarrhea among under-five children.

As Ethiopia is one of the countries where diarrheal morbidity is a significant public health problem [18], further evidence is needed about related factors in regions of the country where up-to-date information has not been available. As part of the efforts to monitor the progress toward achievement of the UN Sustainable Development Goals (SDG) of 2030, specifically to end preventable deaths of newborns and children under five years of age [19], the determinants of diarrhea should be identified so that effective priority intervention areas can be identified to control diarrhea disease.

The incidence of diarrheal diseases varies greatly with a child’s age. The youngest children are most vulnerable to diarrhea because of their immature immune systems [20, 21]. However, lack of evidence for factors significantly associated with acute diarrhea among children aged 0–23 months in Dessie City hinders proper planning for well targeted interventions. Hence, this study was undertaken with the aim of identifying the determinant factors of diarrhea among children under two years of age in Dessie City, northeastern Ethiopia to help to guide the design of appropriate intervention strategies to control the problem diarrhea that area. Furthermore, the study will also show the priority areas among existing intervention areas for resource-wise prevention of childhood diarrhea.

## Methods

### Study setting

The study was conducted in Dessie City, which is located in South Wollo Zone of Amhara Region in northeastern Ethiopia, 401 km north from Addis Ababa. The average monthly minimum and maximum temperatures are 12.4 °C and 26.3 °C, respectively. Dessie City consists of 5 sub-cities comprising 10 urban and 6 rural *kebeles* (A *kebele* is the lowest administrative unit in Ethiopia, with a population of approximately 5000). Based on Ethiopian population projections, Dessie City had a total population of 212,436 in 2014 [22].

### Study design and outcome measurement

A community-based matched case-control study was conducted from January to February, 2018 among cases and matched controls of children aged under two years. Cases were defined as under-two children (0–23 months) with diarrhea during the two weeks before the survey, whereas controls were defined as under-two children without acute diarrhea matched with cases by age and residence (residing in the same *kebele*) during the two weeks before the survey.

Cases were identified based on reporting by a mother/caregiver of a child having had acute diarrhea during the two weeks preceding the survey. World Health Organization (WHO) defined diarrhea as the passage of three or more abnormally loose, watery or liquid stools per day [23]. However, the WHO definition did not specify the recall period and the types of diarrhea as acute, bloody or persistent, and since our study focused on acute diarrhea, we adopted a two-week recall period based on the World Gastroenterology Organization global guidelines for acute diarrhea measurement [24].

Thus, in our study, cases means children with acute diarrhea during the two weeks prior to the survey. The presence or absence of acute diarrhea was assessed by trained data collector based on report by a mother/caregiver using the existing signs and symptoms per the World Gastroenterology Organization global guidelines for acute diarrhea definition “as the passage of three or more abnormally loose, watery or liquid stools per day during the two weeks prior to the survey [24].

### Sample size determination

A matched case-control study sample size determination method using Schlesselman and Stolley sample size estimating equations was used [25], considering 95% confidence level, 80% power. A 15.3% expected proportion of the estimate of the exposure variable “retrieving water from water storage containers with/without handle” for controls was taken from a previous study conducted in Addis Ababa [15]. Expected effects, such as least extreme odds ratio to be detected 2.25, a case-control ratio of 2 and a 10% non-response rate was selected to compensate for sample size reduced by refusal to participate, lack of matched controls for cases and lack of cases that could be paired with controls. Thus, the final sample size was 357 (119 cases and 238 controls).

### Case and matched control selection techniques

First, two of the six rural and five of the ten urban *kebeles* in Dessie City were selected through simple random sampling technique. Then we determined the number of households that had children under two years old from the updated records kept by health extension workers (HEWs) and proportionally allocated them to each *kebele*. Then, during the survey, data collectors identified cases and matched controls using house-to-house transect walks in each *kebel*e until the proportionally allocated sample size achieved. These case and control selection procedures were also briefly explained in another study [16]. During data collection, two matched controls were identified for each case.

During selection, controls were children aged under two years without diarrhea during the two weeks before the survey, matched with cases by age and residence. Matching with residence meant that case and controls should reside within the same *kebele*. Controls were selected after identifying cases. During matching of controls with cases, individual matching by age was used categorized into three age groups: control age = case age ± 2 months for infants (6–11 months), and ± 3 months for toddlers (12–23 months) as explained elsewhere [21, 26]. Age was measured by complete months.

Using a range of ±2 or ± 3 months age for individual matching of control selection meant that a child’s age might intersect another age category, which seemed problematic; we resolved this by considering only these children within the specified age category. However, although the matched age group was wide enough to include overlap when children transition from exclusive to non-exclusive breastfeeding and introduction of complementary foods, this did not compromise our study findings due to the fact that during this case-control study, the associated factors rather than outcome were our interest.

Furthermore, controls were enrolled on the same day as cases identified to increase the effectiveness of the matching since differences in timing of when cases and controls were interviewed might have affected the study, since time-varying environmental factors could have varied and affected cases and controls.

### Household survey data collection and quality control

Data were collected using a pre-tested structured questionnaire and an observational checklist. The questionnaire was first developed in English, and then translated to Amharic (local language) for household survey use. The re-translation of the questionnaire was done from Amharic to English to ensure consistency. The components of the questionnaire were prepared from previously published papers [15, 16, 27], by adapting questions from the 2016 Ethiopia Demographic and Health Survey [EDHS] [13] and the 2006 WHO and UNICEF core questions on drinking water and sanitation for household surveys [28].

The respondents were mothers/caregivers of case and control children. The information of interest (case and control) was obtained about the two weeks prior to the survey. However, some independent variables had a much longer recall period. For instance, asking about exclusive breastfeeding and complementary feeding did not refer only to the prior two weeks. Seven diploma nurses and two environmental health professionals with BSc were recruited as data collectors and supervisors, respectively. Data collectors and supervisors were trained by the principal investigator for three days on the aim of the study, the content of the survey tool, ethical considerations during and after data collection, and approaches to be used during the survey.

The questionnaire was pre-tested on 10% of the sample size in one randomly selected *kebele* (*Arera*) in Dessie City that was not selected for this study. The questionnaires were checked for completeness daily by the principal investigator and supervisors; and households that provided incomplete data were revisited once the same day or the next day to gather the missing data.

### Data management and analysis

Data was entered using EpiData Version 3.1 and exported to STATA Version 14.0 for data cleaning and analysis. Data cleaning was performed using frequency distributions and cross tabulations. Descriptive statistics were used for categorical variables and mean ± SD (standard deviations) for continuous variables.

Conditional logistic regression model was used for data analysis. Bivariate (matched crude odds ratio, [mCOR]) and multivariable analysis (matched adjusted odds ratio, [mAOR]) with 95% confidence interval (CI) was used. From the bivariate conditional logistic regression analysis, variables that had a significance level of *p* < 0.2 was retained for inclusion in the multivariable analysis, then from multivariate conditional logistic regression analysis variables that had *p <* 0.05 were considered as statistically significant and independently associated with acute diarrhea.

## Results

### Socio-demographic characteristics of mothers/caregivers of cases and controls

A total of 119 cases and 238 matched controls of children under the age of two years were included in the study. One hundred thirteen (47.5%) of the mothers/caregivers of the controls and 58 of those of the cases (48.7%) were in the 25–34 age group. The majority of mothers/caregivers of both cases and controls were married, 93 (78.2%) and 214 (89.9%), respectively, while mothers/caregivers of 15 (6.3%) of the controls and 13 (10.9%) of cases were illiterate (Table 1).

**Table 1:** Socio-demographic characteristics of mothers/caregivers of children under two in Dessie City, northeastern Ethiopia, January–February 2018

Variable	Category	Case (***N*** = 119)	Control (***N*** = 238)	Unadjusted mOR (95% CI)	***p***-value
		***n***(%)	***n***(%)		
Age of mother/caregiver (years)	< 25	28(23.6)	85(35.7)	Ref	
	25–34	58(48.7)	113(47.5)	1.65(0.95–2.91)	0.078
	> 34	33(27.7)	40(16.8)	2.76(1.54–7.40)	< 0.001
Respondent relationship to child	Mother	106(89.1)	212(89.1)	Ref	
	Caregiver	13(10.9)	26(10.9)	1.3(0.50–2.00)	0.999
Marital status of mother/caregiver	Single	9(7.6)	12(5.0)	0.57(0.47–2.03)	0.277
	Divorced/widowed	17(14.2)	12(5.1)	2.21(1.46–6.08)	< 0.001
	Married	93(78.2)	214(89.9)	Ref	
Religion	Muslim	53(44.5)	111(46.6)	0.95(0.37–2.49)	0.932
	Christian	66(55.5)	127(53.4)	Ref	
Ethnicity	Amhara	106(89.1)	216(90.8)	0.60(0.18–1.97)	0.397
	Tigre	7(5.9)	15(6.3)	0.62(0.15–2.58)	0.512
	Other^b^	6(5.0)	7(2.9)	Ref	
Mother/caregiver educational attainment	Illiterate	13(10.9)	15(6.3)	2.58(1.10–6.04)	0.029
	Primary	25(21.0)	50(21.0)	1.50(0.78–2.84)	0.223
	Secondary	51(42.9)	85(35.7)	1.78(1.03–3.07)	0.037
	College or above	30(25.2)	88(37.0)	Ref	
Mother/caregiver occupation	Government employee	25(21.0)	48(20.2)	1.06(0.61–1.83)	0.836
	Private employee	18(15.1)	21(8.8)	1.79(0.89–3.59)	0.101
	Hair dresser or barber	10(8.4)	30(12.6)	0.64(0.29–1.39)	0.256
	Housewife	66(55.5)	139(58.4)	Ref	
Educational status of child’s father	Illiterate	10(8.4)	7(2.9)	3.31(1.16–9.43)	0.025
	Primary	19(16.0)	23(9.7)	1.81(0.91–3.63)	0.093
	Secondary	28(23.5)	73(30.7)	0.84(0.49–1.47)	0.548
	College or above	62(52.1)	135(56.7)	Ref	
Occupation of child’s father	Government employee	46(38.6)	107(44.9)	Ref	
	Private employee	24(20.2)	39(16.4)	1.50(0.79–2.76)	0.225
	Merchant	32(26.9)	70(29.4)	1.02(0.60–1.76)	0.929
	Other^c^	17(14.2)	22(9.2)	1.40(0.62–3.15)	0.413
Household monthly income ($ USD)	< $91.33	85(71.4)	191(80.3)	0.59(0.35–1.01)	0.055
	> $91.33	34(28.6)	47(19.7)	Ref	
Number of children under five years old in house	1	107(89.9)	227(95.4)	Ref	
	> 2	12(10.1)	11(4.6)	2.39(0.99–5.75)	0.051
Household size (persons)	< 5	91(76.5)	194(81.5)	0.72(0.41–1.26)	0.245
	> 5	28(23.5)	44(18.5)	Ref	
Occupancy status of the house	Rented	74(62.2)	152(63.9)	0.93(0.58–1.48)	0.749
	Owned	45(37.8)	86(36.1)	Ref	
Number of rooms in the house	1	26(21.8)	47(19.7)	1.36(0.75–2.48)	0.316
	2	41(34.5)	67(28.2)	1.47(0.88–2.43)	0.138
	> 3	52(43.7)	124(52.1)	Ref	

aThe average exchange rate of 1$ USD (United States Dollars) was 27.372 ETB (Ethiopian birr) in January and February 2018

^b^Oromo and Gurage

^**c**^drivers, mechanics, daily laborers

Ref, reference category; Unadjusted mOR, unadjusted matched crude odds ratio; CI, confidence interval

Most of the mothers/caregivers of controls 216 (90.8%) and cases 106 (89.1%) were from the Amhara ethnic group. The religion of 53 (44.5%) cases and 111 (46.6%) controls were Muslim, whereas it was Christians for 66 (55.5%) cases and 127 (53.4%) controls. Mothers/caregivers of more than half 66 (55.5%) of the case and 139 (58.4%) of the control children were housewives (Table 1).

### Case and control child characteristics

Among enrolled children younger than six months, 11 (9.2%) were cases and 24 (10.1%) were controls; and among those 6-11 months 36 (30.3%) were cases and 70 (29.4%) controls; and among those 12-23 months 72 (60.5%) were cases and 144 (60.5%) controls (Table 2).

**Table 2:** Child characteristics among cases and controls in Dessie City, northeastern Ethiopia, January–February 2018

Variable	Category	Case (***N*** = 119)	Control (***N*** = 238)	Unadjusted mCOR (95% CI)	***p***-value
		***n***(%)	***n***(%)		
Child’s age (months)	< 6	11(9.2)	24(10.1)	N/A	
	6–11	36(30.3)	70(29.4)		
	12–23	72(60.5)	144(60.5)		
Child’s sex	Male	60(50.4)	116(48.7)	Ref	
	Female	59(49.6)	122(51.3)	0.93(0.59–1.47)	0.756
Place of child’s birth	At health facility	112(94.1)	229(96.2)	Ref	
	At home	7(5.9)	9(3.8)	0.93(0.59–1.47)	0.365
Exclusive breastfeeding of the child	No	23(19.3)	84(35.3)	2.28(1.42–4.56)	0.002
	Yes	96(80.7)	154(64.7)	Ref	
Current child feeding status	Breast milk with complementary food	78(65.5)	169(71.0)	1.68(0.42–6.68)	0.462
	Complementary food only	44(18.5)	44(18.5)	2.77(0.69–11.2)	0.152
	Breast milk only	10(8.4)	25(10.5)	Ref	
Complementary feeding method of child	With hand	28(23.5)	49(21.9)	2.38(1.16–4.87)	0.018
	Bottle	9(7.6)	7(3.1)	1.46(0.23–9.04)	0.682
	Spoon/cup	17(14.3)	73(32.6)	Ref	
	With all of the above	65(54.6)	95(42.4)	2.73(1.50–4.99)	0.001
Vaccination completed based on the child’s age	Yes	110(92.4)	214(89.9)	Ref	
	No	9(7.6)	24(10.1)	0.70(0.30–1.64)	0.416

N/A, not applicable to analysis since child age was a matching variable

Ref, reference category; Unadjusted mOR, unadjusted matched crude odds ratio; CI, confidence interval

Just over half 60 (50.4%) of cases were male and while females made up a similar proportion of controls half 122 (51.3%). A majority 112 (94.1%) of cases and controls 229 (96.2%) had been born at health facilities. Most of case 96 (80.7%) and control 154 (64.7%) children had been exclusively breastfed for six months after birth. A majority of cases 110 (92.4%) and controls 214 (89.9%) had completed vaccinations scheduled for the child’s age (Table 2).

### Sanitation and hygiene characteristics

All the respondents had access to a latrine and 53 (44.5%) of cases and half 125 (52.5%) of controls had a private household latrine. Feces were observed around the latrine in 44.5% of the case and 57 (23.9%) of control households’ facilities. The proximity of the latrine from home for a majority 98 (82.4%) of case and 184 (77.3%) control households was less than 15 m. More than half 68 (57.1%) the case and control 167 (70.2%) households’ latrines did not have a handwashing facility around the latrine. Mothers/caregivers of fewer than half 51 (42.9%) of cases and fewer than one-tenth 21 (8.8) of controls practiced handwashing at fewer than 3 critical times (Table 3).

**Table 3:** Characteristics of case and control households by sanitation- and water-related factors in Dessie City, northeastern Ethiopia, January–February 2018

Variable	Category	Case (*N* = 119)	Control (*N* = 238)	Unadjusted mOR (95% CI)	***p***-value
		***n***(%)	***n***(%)		
Type of latrine	Private	53(44.5)	125(52.5)	Ref	
	Public	18(15.1)	40(16.8)	1.08(0.56–2.09)	0.782
	Shared	48(40.3)	73(30.7)	1.54(0.95–2.50)	0.451
Feces observed on the floor and/or around the latrine	Yes	53(44.5)	57(23.9)	2.46(1.84–3.93)	< 0.001
	No	66(55.5)	181(76.1)	Ref	
Handwashing facility within/around the latrine	No	79(66.4)	199(83.6)	2.58(1.8–7.89)	0.015
	Yes	40(33.6)	39(16.4)	Ref	
Proximity of the latrine from home (meters)	< 15	98(82.4)	184(77.3)	1.37(0.78–2.40)	0.271
	15 or more	21(17.6)	54(22.7)	Ref	
Garbage/refuse disposal method	Burning	13(13.4)	15(6.3)	1.89(0.82–4.38)	0.134
	Disposed of into open field	10(8.4)	24(10.1)	0.91(0.42–1.96)	0.810
	Taken by house-to-house garbage collectors or put into municipal garbage container	96(80.7)	199(83.6)	Ref	
Domestic sewage discharge method	Open ditch outside the compound	42(35.3)	88(37.0)	2.39(1.52–4.35)	0.004
	Discharged into latrine	25(21.0)	11(4.6)	8.59(3.78–13.5)	0.001
	Discharge everywhere	27(22.7)	23(9.7)	5.74(2.68–12.3)	0.001
	Soak-away pit	25(21.0)	116(48.7)	Ref	
Piped water source type	Public water tap	5(4.2)	13(5.5)	0.75(0.25–2.21)	0.597
	Private water tap	114(95.8)	225(94.5)	Ref	
Mouth size of drinking water storage container(s)	Narrow-mouthed	44(37.0)	62(26.1)	Ref	
	Wide-mouthed	36(30.3)	50(21.0)	0.99(0.56–1.76)	0.989
	Both narrow-mouthed and wide-mouthed	39(32.8)	126(52.9)	0.44(0.26–0.75)	0.002
Method of obtaining water from drinking water storage container	Pouring	19(16.0)	37(15.5)	Ref	
	Dipping	56(47.1)	64(26.9)	1.75(0.90–3.39)	0.099
	Both pouring and dipping	44(37.0)	137(57.6)	0.61(0.32–1.17)	0.138
Daily per capita water consumption (liters)	30 l or more	37(31.1)	100(42.0)	Ref	
	< 30 l	82(68.9)	138(58.0)	1.51(0.98–2.33)	0.065
Mothers/caregivers handwashing practice at critical times	< 3 critical times	51(42.9)	21(8.8)	7.75(4.36–13.10)	< 0.001
	> 3 critical times	68(57.1)	217(91.2)	Ref	

Ref, reference category; Unadjusted mOR, unadjusted matched crude odds ratio; CI, confidence interval

Solid waste disposal for about 96 (80.7%) case and 199 (83.6%) control households was performed by municipal house-to-house garbage collection, whereas 42 (35.3%) of case and 88 (37.0%) of control households discharged their liquid waste into open fields (Table 3).

### Water supply and water handling characteristics

The water source for a majority 147 (95.8%) of case and 225 (94.5%) of control households was a private tap while 5 (4.2%) of case and control 13 (5.5%) households obtained water at a public tap. The water consumption of about two-thirds 82 (68.9%) of cases and 138 (58.0%) controls were less than 30 l per day (Table 3).

More than one-third 44 (37.0%) of cases and 137 (57.6%) of control households used both pouring and dipping methods to get water from a water storage container. However, 19 (16.0%) case and 37 (15.5%) control households used the pouring method only. Water storage containers with a narrow mouth were used by 44 (37.0%) of case and 62 (26.1%) of control households and about one-third 39 (32.8%) of case and half 126 (52.9%) control households used both wide- and narrow-mouthed water storage containers interchangeably (Table 3).

### Multivariable conditional logistic regression analysis

All variables associated with the outcome variable at *p* < 0.2 in the bivariate analysis were entered into multivariable conditional logistic regression. Of the 18 variables that were entered into the multivariable conditional logistic regression, seven variables were found to be independently associated with diarrhea. From the adjusted analysis, diarrhea was independently associated with mothers’/caregivers’ age, marital status, lack of exclusive breastfeeding, presence of feces on the floor and/or around the latrine, no availability of a handwashing facility within/around the latrine, domestic sewage discharge everywhere within and/or outside of compound and handwashing practiced by mothers/caregivers at fewer than three of the critical times.

The odds of developing diarrhea among children under two with mothers/caregivers aged 35 years or older were 2 times greater than those with mothers/caregivers aged 25 years or younger (adjusted mOR = 2.00; 95% CI: 1.37–5.8). Children who had a divorced or widowed mother/caregiver were 1.4 times more likely to develop diarrhea than children of mothers/caregivers who were married (adjusted mOR = 1.40; 95% CI: 1.26–3.30) (Table 4).

**Table 4:** Factors significantly associated with diarrhea among children aged 0–23 months from multivariable conditional logistic regression analysis

Variables	Category	Adjusted mOR (95% CI)	***p***-value
Age of mother/caregiver (years)	< 25	Ref	
	25–34	1.47(0.96–9.24)	0.650
	> 35	2.00(1.37–5.80)	< 0.001
Marital status of mother/caregiver	Single	1.42(0.54–4.40)	0.84
	Divorced/widowed	1.40(1.26–3.30)	0.001
	Married	Ref	
Exclusive breastfeeding of the child	No	2.12(1.15–3.70)	0.002
	Yes	Ref	
Feces observed on the floor and/or around the latrine	Yes	1.37(1.21–3.50)	< 0.001
	No	Ref	
Availability of handwashing facility within/around the latrine	No	1.50(1.30–5.30)	0.030
	Yes	Ref	
Domestic sewage discharge method	Open ditch outside the compound	2.13(1.48–3.70)	0.032
	Discharged into latrine	1.10(0.76–3.80)	0.481
	Discharge everywhere	3.29(1.85–7.50)	< 0.001
	Soak-away pit	Ref	
Mother/caregiver handwashing practice at critical times	< 3 critical times	4.50(2.54–9.50)	< 0.001
	> 3 critical times	Ref	

Ref, reference category; adjusted mOR, adjusted matched odds ratio; CI, confidence interval

The odds of developing diarrhea among the children whose mothers/caregivers washed their hands at fewer than three critical times were 4.5 higher than those whose mothers/caregivers washed their hands at three or more critical times (adjusted mOR = 4.50; 95% CI: 2.54–9.50). The odds of developing diarrhea among under-two children in households that discharged domestic sewage everywhere were 3.29 times greater than those that discharged domestic sewage by the soak-away pit method (adjusted mOR = 3.29; 95%CI: 1.85–7.50) (Table 4).

Under-two children in the households lacking a handwashing facility around the latrine were 1.5 times more likely to have diarrhea than those in households with a handwashing facility around the latrine (adjusted mOR = 1.50; 95% CI: 1.30–5.30). Children in households with visible feces present within/around the latrine were 1.4 times more likely to develop diarrhea than those in households with a clean latrine (adjusted mOR = 1.37; 95% CI: 1.21–3.50) (Table 4). The odds of diarrhea among children under two who were not exclusively breastfed during the first six months of life were 2 times greater than among children who had been exclusively breastfed during the first six months of life (adjusted mOR = 2.12; 95% CI: 1.15–3.70) (Table 4).

## Discussion

This community-based matched case-control study used a matching variable of child’s age and neighbourhood to investigate the main determinants of diarrhea among children aged 0–23 months in Dessie City, northeastern Ethiopia. From the adjusted analysis using conditional logistic regression model, we found that mothers’/caregivers’ age of 35 years or older, widowed/divorced marital status, lack of exclusive breastfeeding of the child, presence of feces on the floor and/or around the latrine, absence of handwashing facility within/around the latrine, discharged liquid waste everywhere within/outside compound mothers'/caregivers' practise of handwashing at fewer than three of the critical times were independently associated with diarrhea among children under two years of age.

Age and marital status of the mothers influenced diarrheal disease [20]. Our findings show that the odds of developing diarrhea were greater among children whose mothers/caregivers were aged 35 years or above. This corroborates a study in Serbo Town in south-western Ethiopia [29] and a study in Burundi [30]. However, this is contrary to studies in Bangladesh [20] and Indonesia [31], which reported that children of mothers/caregivers aged less than 20 years suffered from diarrhea more than those of older mothers. The reason might be that mothers/caregivers 35 years and older spent most of their time on social, cultural and economic activities, events and needs and those younger than 20 years spent more time with their children.

In this study, under-two children living with mothers/caregivers who were divorced/widowed were more likely to get diarrhea than those whose mothers/caregivers were married. These results are similar to those obtained in studies in Jigjiga District, Somali Region, in eastern Ethiopia [32] and in Tehran, Iran [33]. This might be due to the children whose mothers/caregivers were widowed/divorced being more likely to face obstacles and exposure to diarrhea because of the psychological stress and poverty. Female headed households are more likely to experience poverty, impeding their ability to properly care for children.

In our study, lack of exclusive breastfeeding was one of the main factors for diarrhea among children under two years of age. These results were similar to those from other studies in Ethiopia [34–36]. Early initiation of breastfeeding and exclusive breastfeeding were protective against diarrhea in sub-Saharan African countries that had high child mortality due to diarrhea [37]. Exclusive breastfeeding is crucial for children to survive the first months of life because breast milk provides all the nutrients, vitamins and minerals an infant needs for growth and also carries antibodies that help combat disease [7].

Our study shows that children in households that lacked a handwashing facility around the latrine were more likely to have diarrheal disease than those that had a handwashing facility near their latrines. Similar results were reported by studies in Jigjiga Town in Somali Regional State, eastern Ethiopia [38]. According to a WHO report, 88% of diarrhea cases in children under five in developing countries were due to problems with WASH [39]. In our study, we found that 83.6% of the controls and 66.4% of the cases had no handwashing facility around the latrine.

The odds of developing diarrhea were significantly higher among children whose mothers reported washing their hands at fewer than three of the five critical times (before eating, after eating, after cleaning the child, before feeding the child, before preparing food) as compared to children whose mothers reported washing at all five times. These results corroborate those of other studies [36, 40, 41]. The strong link between handwashing at critical times and diarrhea transmission appears to be due to the high risk of hands being exposed to pathogens during the many types of tasks mothers perform every day. The risk of diarrhea transmission is further increased by many mothers/caregivers feeding their children by using their hands. This facilitates pathogen transmission since the infectious agent associated with diarrheal disease is transmitted chiefly under unhygienic conditions. Thus, complementary foods need to be prepared and given to the child in a hygienic manner.

In this study, we found that the presence of feces within/around the latrine increased the odds of diarrhea compared to the presence of hygienic conditions around latrines, similar to studies in Addis Ababa [15] and Bahirdar-Zuria District in northern Ethiopia [40]. Feces around latrine pits may serve as breeding sites of flies that thereby increase the risk of diarrhea [32]. We found households that were not using a proper liquid waste discharge method but defecated in nearby fields to have higher odds of contracting diarrhea. Similar findings were reported by a study in Jigjiga District, Somali Region, eastern Ethiopia [32]. Open-field defecation continues to be widespread in Ethiopia, creating conditions suitable for diarrhea transmission.

### Limitations of the study

Although matching is effective to control confounders at the design stage, there may be a danger in using neighbourhood controls, as similarities of socio-economic status, cultural and behavioral practices and environmental factors might be masking the role of some other potential exposures and relevance as determinants of diarrhea. The other limitation was the use of a two-week recall period, as respondents may be less likely to remember certain practices or exposures, or more likely to report more favorable practices, which create a form of reporting bias during case identification. Therefore, cases may have been affected by recall bias.

We also did not triangulate the acute diarrhea (cases) with the outcome of the health centre records (clinically identified of acute diarrhea), which may affect the correct ascertainment of the presence of diarrhea. However, since we did not study the prevalence of diarrhea, identification of acute diarrhea using a self-report in our study was reasonable due to the fact that we did not generalize the magnitude of acute diarrhea in the study setting. The presence of chronic health conditions, malnutrition status of the child, history of previous episodes, and the duration of exposure to the different risk factors were not considered as potential confounders due to the fact that measuring these variables using self-report might have compromised the generalizability of the study; however researchers are highly encouraged to address this limitation for more inclusive intervention purposes.

The limitations of the study also included lack of objective exposure measurements for fecal indicators, an inability to investigate the magnitude of diarrhea due to the case-control study design nature, and the fact that the study was done only during a dry season (dry season in the study setting was December, January and February), whereas environmental factors might change during the rainy seasons (rainy season period in the study setting was June, July and August). Furthermore, interviewer’s bias may be also considered as a limitation especially during observational data collection for environmental factors, although we handled this by providing training about how to perform data measurement by on-the-spot observation.

Another limitation could be that identification of cause-effect relationship was not addressed in our study due to the study not considering  impact measures. Hence, using a cohort study design and/or randomized control trial studies is highly recommended.

## Conclusion

We conclude that the findings of the study have important implications for prioritizing of health interventions and significant benefits for child health and survival. To prevent diarrheal disease, interventions should prioritize improving practices of exclusive breastfeeding, regular cleaning of latrines, advocating availability of handwashing facilities within/around latrines, using proper domestic sewage discharge methods and improving handwashing practice at the five critical times.

Strengthening awareness and empowerment among mothers/caregivers on environmental hygiene and sanitation practices, especially handwashing habits and domestic sewage discharging methods are essential to preventing diarrhea. Advocating communication to promote hygiene and behavioural change may also raise awareness and empower mothers/caregivers to enhance handwashing practice at critical times. Furthermore, research on determinants of diarrhea among children under two in combination with nutritional status and seasonal variation in the study area is recommended to obtain more concrete evidence.

## Data Availability

Data and all the materials will be available from the corresponding author upon request.

## Abbreviations

mAOR: Matched adjusted odds ratio
mCOR: Matched crude odds ratio
CI: Confidence interval
WHO: World Health Organization

## References

[1] Unger A (2013). Children’s health in slum settings. Arch Dis Child.

[2] Liu L, Johnson HL, Cousens S, Perin J, Scott S, Lawn JE (2012). Global, regional, and national causes of child mortality: an updated systematic analysis for 2010 with time trends since 2000. Lancet.

[3] Riddle MS, DuPont HL, Connor BA (2016). ACG clinical guideline: diagnosis, treatment, and prevention of acute diarrheal infections in adults. Am J Gastroenterol.

[4] Gerald TK, Olivier F, Alok B. et al. Diarrheal diseases. Disease control priorities in developing countries | Gerald T. Keusch, Olivier Fontaine, Alok Bhargava, and others. 2007;371–87.

[5] Christina MS, Blanca O. Diarrheal diseases FACG. 2007.

[6] Walker CLF, Rudan I, Liu L, Nair H, Theodoratou E, Bhutta ZA (2013). Global burden of childhood pneumonia and diarrhoea. Lancet.

[7] WHO (2017). Inheriting a sustainable world? Atlas on children’s health and the environment.

[8] Luby S, Agboatwalla M, Hoekstra R, Rahbar M, Billhimer W, Keswick B (2004). Delayed effectiveness of home-based interventions in reducing childhood diarrhea, Karachi, Pakistan. 2004;71(4):420–27. Am J Trop Med Hyg..

[9] Lule J, Mermin J (2005). E J, Malambo S, downing R, ransom R, et al. effect of home-based water chlorination and safe storage on diarrhea among persons with human immunodeficiency virus in Uganda. Am J Trop Med Hyg.

[10] George CM, Perin J, De Calani KJN, Norman WR, Perry H, Davis TP (2014). Risk factors for diarrhea in children under five years of age residing in peri-urban communities in Cochabamba, Bolivia. Am J Trop Med Hyg.

[11] Mulatya DM, Ochieng C. Disease burden and risk factors of diarrhoea in children under five years: evidence from Kenya's demographic health survey 2014. Int J Infect Dis. 2020;93:359–66 10.1016/j.ijid.2020.02.003 32061860

[12] Bado AR, Susuman AS, Nebie EI (2016). Trends and risk factors for childhood diarrhea in sub-Saharan countries (1990–2013): assessing the neighborhood inequalities. Glob Health Action.

[13] CSA, ICF-International. Ethiopia Demographic and Health Survey 2016. Addis Ababa, Ethiopia, and Calverton, Maryland, USA: Central Statistical Agency [Ethiopia] and ORC Macro2016.

[14] Alebel A, Tesema C, Temesgen B, Gebrie A, Petrucka P, Kibret GD (2018). Prevalence and determinants of diarrhea among under-five children in Ethiopia: a systematic review and meta-analysis. PLoS One.

[15] Adane M, Mengistie B, Kloos H, Medhin G, Mulat W (2017). Sanitation facilities, hygienic conditions, and prevalence of acute diarrhea among under-five children in slums of Addis Ababa, Ethiopia: baseline survey of a longitudinal study. PLoS One.

[16] Adane M, Mengistie B, Medhin G, Kloos H, Mulat W (2017). Piped water supply interruptions and acute diarrhea among under-five children in Addis Ababa slums, Ethiopia: a matched case-control study. PLoS One.

[17] Dagnew AB, Tewabe T, Miskir Y, Eshetu T, Kefelegn W, Zerihun K (2019). Prevalence of diarrhea and associated factors among under-five children in Bahir Dar city, Northwest Ethiopia, 2016: a cross-sectional study. BMC Infect Dis.

[18] MOH (2015). Health Sector Transformation Plan (HSTP) 2015/16–2019/20, The Federal Democratic Republic of Ethiopia Ministry of Health. Addis Ababa.

[19] UN (2016). The Sustainable Development Goals Report 2016. United Nations, New York.

[20] Sarker AR, Sultana M, Mahumud RA, Sheikh N, Van Der Meer R, Morton A (2016). Prevalence and health care–seeking behavior for childhood diarrheal disease in Bangladesh. Glob Pediatr Health.

[21] Kotloff KL, Nataro JP, Blackwelder WC, Nasrin D, Farag TH, S P (2013). Burden and aetiology of diarrhoeal disease in infants and young children in developing countries (the Global Enteric Multicenter Study, GEMS): A prospective, case-control study. Lancet.

[22] CSA. Population Projection of Ethiopia for All Regions At Wereda Level from 2014–2017 Central Statistical Agency 2017.

[23] WHO (2005). The treatment of diarrhea. A manual for physicians and other senior health workers. Department of Child and Adolescent Health and Development.

[24] Farthing M, Salam M, Lindberg G, Dite P, Khalit I, Lindo S, et al. World Gastroenterology Organization Global Guidelines. Acute diarrhea in adults and children: a global perspective. World Gastroenterology Organization; 2012. p. 1–24. 10.1097/MCG.0b013e31826df662 23222211

[25] Schlesselman J, Stolley P (1982). Case-control studies: design, conduct, analysis.

[26] Breurec S, Vanel N, Bata P, Chartier L, Farra A, Favennec L (2016). Etiology and epidemiology of diarrhea in hospitalized children from low income country: a matched case -control study in Central African Republic. PLoS Negl Trop Dis.

[27] Adane M, Mengistie B, Mulat W, Medhin G, Kloos H (2018). The most important recommended times of hand washing with soap and water in preventing the occurrence of acute diarrhea among children under five years of age in slums of Addis Ababa, Ethiopia. J Community Health.

[28] WHO, UNICEF (2006). Core Questions on Drinking Water and Sanitation for Household Surveys. JMP Publication of the World Health Organizations and United Nations Children Fund. Switzerland.

[29] Kasee LF, Merrom OS, Hassa HY. Assessment of the prevalence of diarrheal disease under-five children Serbo town, Jimma zone south West Ethiopia. Clin Mother Child Health. 2018;15(1).1000280.

[30] Diouf K, Tabatabai P, Rudolph J, Marx M. Diarrhoea prevalence in children under five years of age in rural Burundi: an assessment of social and behavioural factors at the household level. Glob Health Action. 2014;7:24895. 10.3402/gha.v7.24895 PMC4141944 25150028

[31] Rohmawati N, Panza A, Lertmaharit S (2012). Factors associated with diarrhea among children under five years of age in Banten Province, Indonesia. J Health Res.

[32] Hashi A, Kumie A, Gasana J (2016). Prevalence of diarrhoea and associated factors among under-five children in Jigjiga District, Somali region, Eastern Ethiopia. Open J Prev Med.

[33] Kolahi AA, NABAVI M, Sohrabi MR (2008). Epidemiology of acute diarrheal diseases among children under 5 years of age in Tehran, Iran. Iran J Clin Infect Dis.

[34] Gizaw Z, Woldu W, Bitew BD (2017). Child feeding practices and diarrheal disease among children less than two years of age of the nomadic people in Hadaleala District, Afar region, Northeast Ethiopia. Int Breastfeed J.

[35] Asfaha KF, Tesfamichael FA, Fisseha GK, Misgina KH, Weldu MG, Welehaweria NB, et al. Determinants of childhood diarrhea in Medebay Zana District, Northwest Tigray, Ethiopia: a community based unmatched case–control study. BMC Pediatr. 2018;18:120. 10.1186/s12887-018-1098-7 PMC5877323 29598815

[36] Getachew A, Guadu T, Tadie A, Gizaw Z, Gebrehiwot M, Cherkos DH, et al. Diarrhea prevalence and sociodemographic factors among under-five children in rural areas of North Gondar zone, Northwest Ethiopia. Int J Pediatr. 2018;2018. 10.1155/2018/6031594. 10.1155/2018/6031594 PMC6008758 29971113

[37] Ogbo FA, Agho K, Ogeleka P, Woolfenden S, Page A, Eastwood J (2017). Infant feeding practices and diarrhoea in sub-Saharan African countries with high diarrhoea mortality. PLoS One.

[38] Bizuneh H, Getnet F, Meressa B, Tegene Y, Worku G. Factors associated with diarrheal morbidity among under-five children in Jigjiga town, Somali Regional State, eastern Ethiopia: a cross-sectional study. BMC pediatrics. 2017;17:182. 10.1186/s12887-017-0934-5 PMC5568275 28830462

[39] WHO (2013). End preventable deaths: Global Action Plan for Prevention and Control of Pneumonia and Diarrhoea.: World Health Organization/The United Nations Children’s Fund (UNICEF).

[40] Asnakew DT, Teklu MG, Woreta SA (2017). Prevalence of diarrhea among under-five children in health extension model households in Bahir Dar Zuria district, North-Western Ethiopia. Edorium J Public Health.

[41] Regassa W, Lemma S (2016). Assessment of diarrheal disease prevalence and associated risk factors in children of 6-59 months old at Adama District rural Kebeles, eastern Ethiopia, January/2015. Ethiop J Health Dev.

[42] WMA (2008). World Medical Associations Declaration of Helsinki. Ethical principles for medical research involving human subjects, 6th revision. Helsinki, Finland.

